# The Semmelweis Framework: structuring evidence foundations and expert opinion in clinical practice guidelines

**DOI:** 10.1186/s12874-026-02938-6

**Published:** 2026-07-18

**Authors:** Blin Nagavci, Margaret Renn Andrews, Jens Vikse, Paul Studenic, Tanja Stamm, Botond Lakatos

**Affiliations:** 1 Doctoral School of Clinical Medicine, Semmelweis University, Budapest, Hungary; 2 Institute for Outcomes Research, Center for Medical Data Science, Medical University of Vienna, Vienna, Austria; 3 Ludwig Boltzmann Institute for Arthritis and Rehabilitation, Vienna, Austria; 4 Department of Rheumatology, Stavanger University Hospital, Stavanger, Norway; 5 Department of Clinical Science, University of Bergen, Bergen, Norway; 6 Department of Chemistry, Bioscience and Environmental Engineering, University of Stavanger, Stavanger, Norway; 7 Department of Internal Medicine 3, Division of Rheumatology, Medical University of Vienna, Vienna, Austria; 8 Department of Hematology and Internal Medicine, Division of Infectious Diseases, Semmelweis University, Budapest, Hungary; 9 National Institute of Hematology and Infectious Diseases, South Pest Central Hospital, Budapest, Hungary

**Keywords:** Expert opinion, Guidelines, Methodology, Transparency

## Abstract

**Background:**

For many guideline-developing organisations, expert opinion remains essential when evidence is scarce. However, its unstandardised use remains a significant gap in guideline methodology. This article presents the development of the Semmelweis Framework, a methodological tool for structuring expert opinion in clinical practice guidelines when evidence is limited, indirect, or absent.

**Methods:**

The Semmelweis Framework was developed through a stepwise methodological process. First, a meta-epidemiological study mapped expert opinion use across 2,296 recommendations from 136 organisations. Second, a methodological review of 98 guideline manuals synthesised the functional definition of expert opinion that informed the Framework. Building on this definition, the Framework was refined through independent multi-reviewer application to 30 published recommendations, with calibration meetings, qualitative comparison of classifications, and parallel development of a Preconditions Checklist.

**Results:**

The Semmelweis Framework offers an approach for structuring the role of expert opinion in clinical guidelines by classifying recommendations in four classes according to their evidentiary foundation. These classes form a continuum: from recommendations based on direct comparative evidence to those relying entirely on expert opinion when no relevant group-level evidence exists. Class I is grounded in direct comparative evidence. Class II addresses indirect evidence and requires explicit labelling of indirectness while using expert opinion to support applicability. Class III relies on non-comparative evidence and is heavily influenced by expert opinion, whereas Class IV is based entirely on expert opinion, operationalised as structured expert judgement, when no group-level evidence exists. Across all classes, fixed verbs and notations are proposed to standardise communication. A core component of the Framework is the specifically developed Preconditions Checklist, designed to ensure integrity and transparency when recommendations rely substantially on expert opinion. Under the Semmelweis Framework, “expert opinion” should not be used as a generic recommendation label; instead, its roles are redistributed across recommendation classes, each with explicit foundations and labelling.

**Conclusions:**

The Semmelweis Framework reframes the role of expert opinion and offers an approach for structuring its use in clinical guidelines. By introducing safeguards and new recommendation classes according to their evidentiary foundations, the Framework addresses a long-standing methodological gap.

**Supplementary Information:**

The online version contains supplementary material available at https://doi.org/10.1186/s12874-026-02938-6.

**Table:** 

Contributions to the literature
• The Semmelweis Framework operationalises the use of expert opinion in guideline development through a structured classification system.
• It introduces structured safeguards when guidance is issued without strong evidence, supporting accountability, documentation, and justification.
• It bridges traditional grading systems and GRADE by offering a pragmatic, rigorous solution for guideline developers facing scarce or absent evidence.
• It redistributes the roles traditionally grouped under “expert opinion” across explicitly defined recommendation classes.

## Background

Clinical guidelines play a pivotal role in standardising care, enhancing clinical decision-making, and ensuring that practices are evidence-based. To achieve this, guidelines should be developed through a systematic review and the use of the best available evidence [1]. However, when evidence is scarce or inconclusive, guideline panels often face a dilemma on how to bridge such evidence gaps and ensure actionable recommendations. For many guideline-developing organisations, expert opinion remains essential in these scenarios [2–7]. Previous studies have shown that 66% of national and international guideline-developing organisations utilise expert opinion for issuing recommendations, mainly when evidence is lacking [2]. This underscores the important role of expert opinion in the development of clinical guidelines, contradicting the notion that expert opinion should be excluded from this process. When empirical evidence is limited or absent, guideline panels may need to draw on expert opinion as a source of practical guidance. Such use should be explicit and transparent, allowing users to understand the foundation of the recommendation and assess its validity [8, 9]. However, how expert opinion should be defined, applied, and presented remains unstandardised and controversial.

This issue stems primarily from a fundamental lack of widely accepted consensus on what expert opinion truly means in a guideline. Guideline-developing organisations conceptualise expert opinion in markedly different ways, applying it to a wide spectrum of foundations, from clinical experience and mechanistic reasoning to indirect and low certainty evidence [2]. This leads to substantial differences in how expert opinion is utilised across organisations and their guidelines.

Complicating the issue further, evidence grading systems treat expert opinion inconsistently, leading to two main schools of thought. On the one hand, traditional evidence-grading systems classify evidence primarily by study design and often place expert opinion at the lowest level of the evidence hierarchy [10–12]. In contrast, Grading of Recommendations, Assessment, Development and Evaluation (GRADE), the most widely adopted system (used by 53% of guideline-developing organisations globally) [2], takes an entirely different philosophical position. It does not recognise expert opinion as a distinct entity; instead, it defines it as the process of interpreting evidence through the lens of expert experience and knowledge [13]. Both schools of thought have their methodological shortcomings. Traditional grading systems, although they allow the use of expert opinion, often do not distinguish it from very low-certainty evidence and fail to capture the full range of roles and foundations on which it rests [2, 3]. On the other hand, GRADE’s approach does not always align with the practical needs of guideline developers. Among guideline-developing organisations worldwide that use GRADE, 42% still rely on expert opinion when issuing recommendations in situations of scarce evidence [2], even though GRADE does not formally support expert opinion as a separate basis for recommendations. When such input is used without explicit labelling, its role may become obscured: recommendations may be presented as supported by low or very low certainty evidence or incorrectly labelled as Good Practice Statements. In such cases, guideline users may struggle to discern the true foundation of a recommendation and the extent to which expert opinion contributed to its formulation [14–17].

Altogether, the diversity in how expert opinion is defined, applied, and presented reflects a serious gap in guideline development methodology, and highlights a need for further guidance and standardisation. The Semmelweis Framework was developed as a structured approach, with built-in safeguards, to support more transparent and consistent use of expert opinion in guideline development. The aim of this article is to describe the development of the Semmelweis Framework and its accompanying implementation tools.

## Methods

### Overview of the Framework development

The Semmelweis Framework was developed through a stepwise methodological process. The author team included clinicians and guideline methodologists with experience in clinical practice guideline development. First, prior empirical work was used to characterise the practical problem of expert opinion use in clinical practice guidelines [3]. Second, a methodological review of 98 guideline manuals provided the foundational definition of expert opinion on which the Framework was built [2]. Third, this definition was translated into an initial classification system for recommendations. Finally, the Framework and its accompanying tools were refined through independent multi-reviewer application to published recommendations, followed by calibration meetings and qualitative discussion.

### Empirical mapping of expert opinion use

A meta-epidemiological study assessed patterns of expert opinion use in clinical guidelines by evaluating 2,296 clinical recommendations issued by 136 organisations [3]. This study provided an analytical map of the magnitude and nature of the problem in one clinical field. The conceptual foundation of the Framework, however, was derived from the subsequent methodological review of 98 guideline manuals from guideline-developing organisations across clinical fields [2].

### Foundational methodological review and definition of expert opinion

Given the lack of a widely accepted definition of expert opinion in clinical guidelines, a methodological review was conducted in which 98 methodological manuals from guideline-developing organisations were analysed [2]. This review led to the synthesis of a comprehensive definition of expert opinion, which underpins the Semmelweis Framework:

*“The concept of expert opinion in clinical guidelines refers to the synthesis of guidance derived from clinical experience*,* expert judgment*,* indirect evidence*,* very low-quality evidence or mechanism-based reasoning*,* aimed at supporting clinical decision-making in the absence of evidence or when inconclusive evidence requires contextualization and interpretation”* [2].

Building on this definition, expert opinion was conceptualised as a broad, composite construct. It encompasses several distinct foundations, including clinical experience, mechanistic reasoning, indirect evidence, and very low-certainty evidence.

### Conceptual development of the Framework

The conceptual development of the Framework followed a deductive process based on the foundations of expert opinion identified in the methodological review of guideline manuals [2]. These foundations were used as the starting categories for defining recommendation classes in which expert opinion contributes substantially to the evidentiary basis or interpretation. Because the Framework was intended to classify the full spectrum of guideline recommendations, including those based primarily on empirical evidence, direct comparative evidence was included as a separate class and became Class I recommendations. Indirect comparative evidence was assigned to Class II. Group-level evidence without a concurrent comparator was assigned to Class III. Clinical experience, mechanism-based reasoning, case reports, case series, and collective panel judgement were grouped under Class IV.

### Iterative refinement using published recommendations

To further develop and refine the Framework, a qualitative iterative refinement exercise was conducted using published recommendations from existing clinical guidelines. Data extraction sheets were developed and piloted to support this process. These sheets captured general information about the guideline source, the original recommendation text, the grading system used, the rationale provided by the original authors for the issued recommendation, and the primary studies cited. For piloting, three reviewers independently applied the sheets to two sample recommendations from two guidelines. A calibration meeting was then held to resolve discrepancies and refine the extraction tool.

For the full iterative refinement exercise, a sample of recommendations was drawn from a previously published list of guideline-developing organisations [2]. Using a computer-generated random sequence (https://www.calculator.net/), the lead methodologist assigned five organisations to each of three initial reviewers (out of five reviewers available). Each reviewer then selected a recent guideline (published in the last 5 years) per assigned organisation and two recommendations from each guideline; one recommendation explicitly labelled as expert opinion, consensus-based, or supported by very low-certainty evidence, and one recommendation supported by higher-certainty evidence. In total, 15 clinical guidelines and 30 recommendations were selected. The aim was to sample a variety of recommendations from different organisations, medical fields, topics and methodologies.

The 30 recommendations (listed in Additional file 1) were compiled into an Excel sheet, shuffled and randomly distributed among the five reviewers using the same randomisation tool. Each recommendation was assessed independently by three of the five reviewers, who applied the current version of the Semmelweis flowchart using the piloted extraction sheets. Reviewers did not have access to each other’s extractions or classifications before the calibration meetings. For each recommendation, reviewers: (i) read the rationale of the recommendation provided by the guideline authors; (ii) examined the underlying evidence and identified the study type; (iii) applied the Semmelweis Framework and assigned the most appropriate class; (iv) rephrased the recommendation to align with the Framework structure (without changing its intended direction); and (v) recorded any issues or suggestions for improvement. No de novo evidence synthesis, effect estimation, or re-assessment of risk of bias/certainty of evidence was undertaken as part of the refinement exercise.

For each batch of three to five recommendations, the review team met online (nine meetings in total), during which the extraction sheets for each recommendation were presented. The entire team discussed the source recommendation, its evidence foundation and compared the extractions and classifications. The three reviewers who extracted the data also presented their experience and discussed reasons for divergent judgements and identified ambiguities in the decision rules. Issues that emerged from these discussions were used to revise the flowchart and accompanying text before the next batch of recommendations was assessed. Through this iterative process, several intermediate versions of the classification system were produced and refined. After the main conceptual revisions had been completed, the final version of the Framework was applied to the last nine recommendations. No further conceptual changes were considered necessary after this final application; only minor wording edits were made to improve clarity of the flowchart and accompanying text.

As a result, no single stable version was applied to all recommendations. Because the primary aim of this exercise was to develop and refine the Framework rather than to test a fixed instrument, formal quantitative measures of agreement, such as kappa statistics, were not calculated because they would not have been meaningful in this development phase. Agreement and usability were therefore assessed qualitatively through iterative discussions, rather than through formal statistical analysis.

### Development of the preconditions checklist

The Preconditions Checklist (required for Classes II-IV) was developed iteratively in parallel with the iterative refinement exercise. Draft preconditions were compiled a priori based on the prior work defining the concept of expert opinion [2], and were subsequently refined based on issues identified during extraction and class assignment in the iterative refinement (e.g., ambiguity regarding the evidence foundation, insufficient justification for issuing guidance, or unclear documentation of the reasoning). After each calibration meeting, items were revised when gaps were identified and reworded to ensure feasibility and consistent application. The Checklist was finalised when repeated application no longer revealed major conceptual gaps and only minor wording edits were required.

### Reporting considerations

No reporting guideline directly applicable to methodological framework development was identified. Therefore, no formal reporting guideline was used for this manuscript. Instead, the Methods section was structured to transparently describe the development process, including empirical mapping, the foundational methodological review, conceptual development, iterative refinement using published recommendations, and development of the Preconditions Checklist.

The use of AI-assisted technologies is reported in the Declarations section.

## Results

### The Semmelweis Framework: structuring evidence foundations and expert opinion in clinical practice guidelines

The Framework is named after Semmelweis University, where this work originated. The Semmelweis Framework is a methodological umbrella framework that uses evidence as a starting point to differentiate between classes of recommendations. Unlike grading systems that classify evidence mainly by study design or certainty, the Framework classifies recommendations according to the extent to which the body of evidence directly and appropriately supports the recommendation. The core philosophy of the Semmelweis Framework is therefore to differentiate bodies of evidence for each recommendation, structure expert input through clearly defined criteria, and use explicit labelling to ensure that guideline users understand the foundation of each recommendation. This additional structuring is intended to address a gap that may remain when GRADE is applied alone.

Accordingly, the Framework is intended to complement, not replace, GRADE. Like GRADE, it starts from a systematic evidence review, retains the distinction between certainty of evidence and strength of recommendations, and uses standard GRADE certainty ratings for recommendations based on comparative evidence. Its main difference is that it adds a structured classification of recommendations according to their evidentiary foundation: direct evidence, indirect evidence, non-comparative evidence, or expert judgement. This structure introduces specific labels and wording rules for each class, as well as additional safeguards when recommendations rely on indirect, non-comparative, or judgement-based foundations, while preserving the main GRADE process.

The Framework is intended for clinical practice guidelines that provide recommendations in the domains of treatment, diagnosis and prevention. While applicable across the full spectrum of evidence, it is particularly useful in situations where evidence is limited, indirect, or absent. This added value is particularly relevant in low-evidence contexts, such as rare diseases, where guideline developers often need to combine scarce or very low-certainty evidence with indirect evidence, registry data, mechanistic reasoning, and expert-based judgement, while still making the basis of recommendations transparent [18].

#### The term “Expert Opinion”

The term expert opinion is retained in this article to describe the overarching construct analysed in previous work and to maintain alignment with existing guideline manuals of guideline-developing organisations. Under the Semmelweis Framework, however, expert opinion should not be used as a generic label for individual recommendations or their foundations. Instead, the roles traditionally grouped under expert opinion are redistributed across explicitly defined recommendation classes according to the underlying evidentiary foundation.

#### Applying the Semmelweis Framework

The Semmelweis Framework is applied after the standard evidence review process has been completed. The guideline panel should first conduct the systematic review, including systematic literature searches, study selection, data extraction, risk-of-bias assessment, analysis and synthesis of the data, and certainty-of-evidence assessment using GRADE. These standard steps are performed according to existing methodological guidance and are not modified by the Semmelweis Framework.

**Fig. 1:**
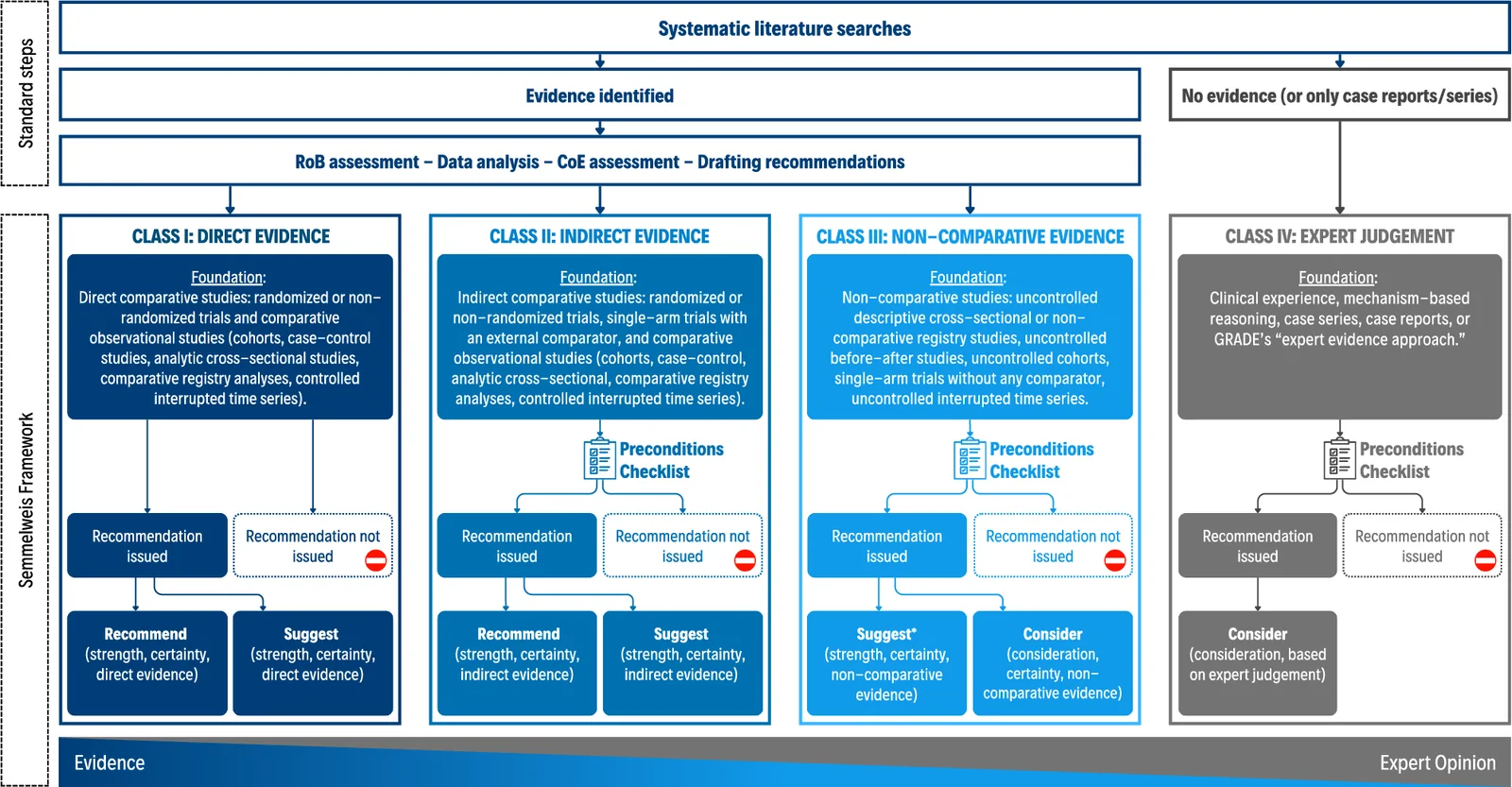
Flowchart of the Semmelweis Framework classification process. RoB: Risk of Bias, CoE: Certainty of Evidence. *Conditional recommendations in Class III should be reserved only for exceptional cases; the default strength in this class is “Consideration”

Once the evidence base has been reviewed, the panel applies the Semmelweis Framework when formulating each recommendation. The first step is to determine the evidentiary foundation that most directly supports the recommendation (Fig. 1). If direct comparative evidence addressing the recommendation question is available and is the main basis for decision-making, the recommendation is classified as Class I. If the recommendation relies mainly on comparative evidence that is indirect because of important differences in population, intervention, comparator, outcome, or setting, it is classified as Class II. If the recommendation is based on group-level, non-comparative evidence, it is classified as Class III. If no relevant group-level evidence is available, or if only case reports, case series, mechanism-based reasoning, or the panel’s collective clinical experience are available, the recommendation is classified as Class IV.

The Semmelweis class is assigned at the level of each individual recommendation, not at the level of the research question. A single research question may therefore generate several recommendations that fall into different classes. For example, a main recommendation on the first-line use of an intervention may be classified as Class I if supported by direct comparative evidence, whereas an additional recommendation on timing, monitoring, adherence, or a rescue strategy may be classified as Class IV if based only on expert judgement (Fig. 1).

#### Description of recommendation classes

The Semmelweis Framework defines four distinct classes of recommendations, each reflecting a different evidentiary foundation and the corresponding role of expert opinion. These classes form a structured continuum: from recommendations based on direct comparative evidence to those relying entirely on expert judgement in the absence of relevant group-level evidence. As the certainty and directness of evidence decrease across the classes, the influence of expert opinion correspondingly increases.

#### Class I: Recommendations based on direct evidence

Class I includes recommendations grounded in comparative evidence that directly addresses the research question. The evidence forming the basis of Class I recommendations typically includes comparative studies, such as: randomized or non-randomized trials, and comparative observational studies (cohorts, case-control studies, analytic cross-sectional studies, comparative registry analyses, controlled interrupted time series), or systematic reviews of such studies.

*Strength and notations*: Recommendations in Class I may be issued as either strong or conditional, according to the GRADE guidance [19]. Strong recommendations (“recommend” or “recommend against”) are generally supported by high or moderate certainty evidence, where the balance of benefits and harms is clear and the intervention is considered acceptable and feasible. Conditional recommendations (“suggest” or “suggest against”) are typically based on lower certainty of evidence, or when the balance of benefits and harms is context-dependent, varies across settings, or may be influenced by patient values and preferences. As an additional layer to GRADE notations, the notation “direct” needs to be added in brackets, i.e. “moderate certainty direct evidence” (Table 1).

**Table 1:** Classification of recommendation classes and accompanying statements with examples

Classes	Strength	Verb	Notation	Examples with verbs and notations
**Class I**: Direct evidence from comparative studies	Strong	Recommend	Direct evidence	We recommend … / We recommend against… (strong recommendation, high certainty direct evidence).
	Conditional	Suggest	Direct evidence	We suggest … / We suggest against … (conditional recommendation, low certainty direct evidence).
**Class II**: Indirect evidence from comparative studies	Strong	Recommend	Indirect evidence	We recommend … / We recommend against… (strong recommendation, moderate certainty indirect evidence).
	Conditional	Suggest	Indirect evidence	We suggest … / We suggest against … (conditional recommendation, low certainty indirect evidence).
**Class III**: Non-comparative evidence	Conditional*	Suggest	Non-comparative evidence	We suggest … / We suggest against … (conditional recommendation, very low certainty non-comparative evidence).
	Consideration	Consider	Non-comparative evidence	Consider … / Consider avoiding … (consideration, very low certainty non-comparative evidence).
**Class IV**: Expert judgement	Consideration	Consider	Based on expert judgement	Consider … / Consider avoiding … (consideration, based on expert judgement*).*
**No recommendation**	No recommendation could be made for or against … due to lack of evidence (no recommendation, no evidence). No recommendation could be made for or against … due to inconclusive evidence (no recommendation, inconclusive evidence). No recommendation could be made for or against … due to lack of consensus (no recommendation, lack of consensus).			

*Conditional recommendations in Class III should be reserved only for exceptional cases. The default strength in this class is “Consideration”

*Role of expert opinion in this class*: When the evidence is of high or moderate certainty, the evidence itself is usually sufficient to support a recommendation, therefore expert opinion has a very limited role. However, when the certainty is low or very low, some degree of interpretation is necessary to reach a conclusion. In such cases, expert opinion may assist in contextualising the findings, but it is not the primary foundation of the recommendation. Therefore, while expert opinion may play a supporting role in the interpretation of low certainty evidence, recommendations in this class remain firmly rooted in direct, comparative evidence.

#### Class II: Recommendations based on indirect evidence

Class II recommendations rely primarily on indirect evidence, when direct evidence is not available. The evidence is considered indirect due to differences in any of the PICO elements (population, intervention, comparator, outcome, or setting) between the available studies and the recommendation question, according to the GRADE guidance [20]. This evidence may come from comparative studies, such as randomized or non-randomized trials, single-arm trials with a historical or external comparator, and comparative observational studies (cohorts, case-control studies, analytic cross-sectional studies, comparative registry analyses, controlled interrupted time series), or systematic reviews of such studies.

In practice, potential indirectness should be considered when the review question, search strategy, and inclusion criteria are being developed, rather than only after the evidence has been retrieved and screened. Guideline panel members often have prior knowledge of the type of evidence likely to be available. This knowledge may be supplemented by a preliminary scoping search in one major bibliographic database, before running main literature searches, to identify whether direct evidence is likely to exist and whether adjacent but clinically relevant evidence may need to be considered. These inputs should be used to decide whether evidence from closely related populations, interventions, comparators, outcomes, or settings may be needed to inform the recommendation. When direct evidence is expected to be scarce or absent, the protocol and inclusion criteria should explicitly define which forms of indirect evidence will be considered acceptable, so that relevant evidence is not missed during study selection. Such evidence may be used when the panel judges it sufficiently applicable to the target recommendation question, and the nature of the indirectness should be clearly documented.

To make this decision, the guideline panel must consider two key aspects about the evidence base:

1. The nature of indirectness: If there are clinically important differences in population, intervention, comparator, setting or outcomes between the available evidence and the guideline question.
2. The balance of evidence: Whether the information that primarily drives the recommendation comes from indirect rather than direct evidence. This judgement should not be based only on the number of studies, but on the overall contribution of the evidence, including the number of participants and events, precision of the estimates, methodological limitations, consistency of findings, importance of the outcomes, and clinical relevance to the recommendation question.

In some cases, a body of evidence may be considered direct for certain outcomes and indirect for others. In such situations, when outcome-level directness is mixed, the classification should be based on the outcome(s) that most strongly drive the recommendation. If the decision relies primarily on indirect outcomes, or if these dominate the evidence base in terms of weight or importance, the recommendation should be classified as Class II. There are no specific thresholds for such decision in this Framework; this remains a matter of panel judgement, determined through discussion on a case-by-case basis.

*Strength and notations*: Recommendations in this class are usually conditional. However, in cases where this is deemed appropriate, strong recommendations may be issued according to GRADE guidance [19]. The verbs to be used are “suggest/suggest against”, or “recommend/recommend against”. The notation “indirect” needs to be added to the standard notation, e.g.: “low certainty indirect evidence” (examples in Table 1).

*Role of expert opinion in this class*: Expert opinion plays a larger role in this class. The panel first needs to decide which available studies are sufficiently applicable to be considered indirect evidence. The interpretation of this evidence, and the applicability of conclusions to the target question, are therefore influenced by expert opinion.

#### Class III: Recommendations based on non-comparative evidence

Class III recommendations are based on studies involving a defined group of subjects, without a separate concurrent control group. Evidence sources for this class include uncontrolled descriptive cross-sectional or non-comparative registry studies, uncontrolled before-after studies, uncontrolled cohorts, single-arm trials without any comparator and uncontrolled interrupted time series. Case series/reports do not belong in this class.

*Strength and notations*: Due to the lack of a comparative study design to answer a research question, evidence in this class has considerable limitations. Therefore, recommendations in this class are generally expressed as “considerations” using the verb “consider/consider avoiding”. In exceptional cases where the magnitude of effect is large or there is an imminent risk of severe harm (e.g., clear safety signal), the panel may issue a conditional recommendation using the verb “suggest/suggest against”. In both cases, the notation “non-comparative evidence” must be used (examples in Table 1). Strong recommendations are not warranted in this class.

*Role of expert opinion in this class*: Because the available evidence lacks a comparative design, expert opinion has a major role in interpreting the findings and determining their applicability to the recommendation.

#### Class IV: Recommendations based on expert judgement

The foundations for this class are the panel’s clinical experience, mechanism-based reasoning and sources such as case series and case reports. The panel’s experience could be harnessed through discussions or collected through a more structured method such as GRADE’s “expert evidence” approach [21, 22]. As no group-level comparative evidence is available, formal risk-of-bias assessment and certainty of evidence assessment are not applicable for this class.

*Strength and notations*: Recommendations in this class are framed as considerations, clearly indicating their reliance on professional and collective judgement of the panel rather than empirical data. Therefore, recommendations should be phrased with verbs “consider” or “consider avoiding” and must include the notation: “based on expert judgement” (examples in Table 1). Conditional and strong recommendations are not warranted in this class.

*Role of expert opinion in this class*: Class IV is based entirely on expert opinion, operationalised as structured expert judgement, when no group-level evidence exists.

#### When no recommendation can be made

If no recommendation is made by the guideline panel, this must be explicitly stated, with the reason, for example: due to inconclusive evidence, lack of evidence or lack of panel consensus (Table 1).

#### The preconditions checklist

Classes II–IV require completion of the Preconditions Checklist before a recommendation is issued (Additional file 2). The Checklist is intended to act as a safeguard for recommendations that rely on less direct or judgement-dependent foundations and are therefore substantially influenced by expert opinion. Its purpose is not to prevent expert-judgement-based recommendations, but to ensure that the panel has considered and documented the key conditions that justify issuing such guidance.

The Checklist prompts guideline panels to document why a recommendation is needed, to confirm that direct comparative evidence is absent or insufficient, and to make explicit the reasoning used to formulate the recommendation. Many of its items reflect considerations that guideline panels would normally discuss when moving from evidence to recommendations. The Checklist should therefore be used as a documentation and consistency tool.

For a recommendation to be issued in Classes II–IV, all preconditions in the checklist should be met. If they are not met, the panel should avoid issuing a recommendation with an insufficiently justified foundation and should instead summarise the available evidence, explain why no recommendation could be made, and, where appropriate, formulate a research recommendation.

#### Certainty of evidence labelling

The Framework uses the standard GRADE notations for Classes I and II (“very low”, “low”, “moderate” and “high”). In addition, the evidentiary foundation of each recommendation must be explicitly labelled. Class I recommendations are labelled “direct evidence”, while Class II recommendations are labelled “indirect evidence”. The Framework also introduces a new notation for Class III, where recommendations are labelled “non-comparative evidence”, while Class IV recommendations are marked as “expert judgement”, a term that is more suitable and reflects the true nature of such recommendations (Table 1).

#### Remarks or contextual notes

The Framework encourages using remarks below each recommendation, to supplement the recommendations by clarifying the scope, implementation, or practical implications. They might also articulate already established common clinical practices. Remarks may be based on the literature or on the panel’s collective expertise. To preserve transparency, remarks should clearly indicate their underlying basis: when they refer to specific evidence, they should include appropriate citations, and when they are primarily based on expert judgement or common practice, this should be explicitly stated in brackets (e.g. “expert judgement” or “common practice”). More importantly, they need to be clearly delineated from the official recommendations, so users do not mistake them as such (Table 2).

**Table 2:** Types of remarks with examples

	Notation	Examples
Remark types	Reference	Evidence suggests that … in cases … (reference).
	Common practice	It is common practice to … in cases … (common practice).
	Expert judgement	The panel considered that … may be relevant in cases … (expert judgement)

#### Implementation resources

To facilitate implementation, the Semmelweis Framework is supported by a set of practical tools, including (i) a decision flowchart to classify each recommendation based on the foundation available (Fig. 1), (ii) a classification table summarising the requirements, phrasing, and role of expert opinion for each class (Table 1), (iii) a Preconditions Checklist to ensure methodological safeguards are met (Additional file 2), and (iv) a frequently asked questions (FAQ) guide, addressing common implementation issues (Additional file 3).

## Discussion

### Summary

The Semmelweis Framework offers a method for structuring the relationship between empirical evidence and expert opinion, supported by safeguards. It defines four recommendation classes based on the type and directness of evidence.

### Implications for guideline development

The most significant conceptual shift introduced by the Semmelweis Framework is the explicit categorisation of expert opinion into distinct recommendation classes, each with defined foundations and conditions for use. Two safeguards operationalise this approach: the explicit classification and labelling, and the Preconditions Checklist.

The Framework also introduces “considerations” for guidance based on weaker foundations (Classes III-IV), to signal a cautious interpretation. This may help curb the documented tendency to issue strong recommendations despite low or very low certainty evidence, an issue affecting up to half of all strong recommendations in GRADE-based guidelines [17, 23, 24].

The Framework also clarifies that “consensus” describes agreement among panel members and should not be used as a proxy for either expert opinion or evidentiary foundation, an issue frequently observed in this field [3, 16, 25].

Another important implication is how indirect evidence is approached in the Framework. In the GRADE approach, indirectness is a reason to downgrade the certainty of evidence within a single, four-tiered scale. A recommendation based on evidence with substantial indirectness may receive the same certainty rating as one based on direct evidence, yet their interpretation is vastly different. The Semmelweis Framework diverges from this approach when indirectness is so substantial that it becomes the central basis for issuing a recommendation. Such recommendations are labelled as Class II (indirect evidence), while preserving all GRADE principles for evidence assessment. This ensures that when relying entirely on extrapolation from indirect evidence, it is made explicit to the end-users.

The Framework also has implications for the use of GRADE’s Good Practice Statements. This approach was introduced by GRADE as a narrow, exceptional category for situations in which an action is clearly necessary or beneficial, and where a formal systematic review would add little to decision-making relative to the effort required [26, 27]. The Semmelweis Framework does not aim to redefine their original purpose. However, it offers an alternative approach for most scenarios in which Good Practice Statements have been misused in practice [15]. The Framework enables guideline developers to issue expert-input driven guidance in a structured and accountable way, and in doing so, it reduces the need to rely on Good Practice Statements as a catch-all solution. This may limit their use to the appropriate circumstances for which they were originally intended.

In addition, the Framework provides a structured format for applying GRADE’s recently introduced “expert evidence approach” [21, 22], by classifying those recommendations under Class IV (expert judgement). This avoids their misrepresentation as evidence-based guidance.

### Strengths

The main strength of the Semmelweis Framework lies in its design to address the long-standing challenge of utilising expert opinion in guideline development. It offers a practical and transparent solution for harnessing expert input in clinical guidelines, with a structured Checklist as an additional safety layer. To the best of our knowledge, this is the first Checklist developed specifically for this purpose. As part of this structure, the Framework is intended to reduce reliance on vague or inconsistently applied terms, such as “expert opinion” or “consensus” recommendations, replacing them with clearly defined evidentiary foundations and action verbs.

The Framework’s relationship with GRADE is another key strength. It acknowledges its critical and irreplaceable role in developing trustworthy guidelines and is designed to be used alongside it rather than to replace it. GRADE already offers important concepts for handling situations with limited or indirect evidence, including Good Practice Statements and the “expert evidence approach” [21, 22, 27]. However, GRADE does not explicitly distinguish recommendation classes by their evidentiary foundations, as its four-level certainty rating was not intended for this purpose [13]. The Semmelweis Framework therefore complements GRADE by adding explicit labelling for foundations. In this way, it provides a transparent option to guideline-developing organisations that have traditionally modified GRADE to issue expert opinion recommendations in situations where evidence is scarce or absent.

Conceptually, the Framework bridges traditional grading systems, which explicitly recognise expert opinion, and the GRADE approach. The Semmelweis Framework draws on the former by recognising expert opinion as a distinct construct and redistributing its roles across recommendation classes, while embedding the concept within a GRADE-compatible structure. This provides a common structure and terminology that might support harmonisation across guideline-developing organisations. Finally, the Framework is accompanied by implementation tools (decision flowchart, classification table, Preconditions Checklist, and FAQ) to support consistent and practical use.

### Limitations

Several limitations must be noted. First, the Framework has been developed through a qualitative and iterative refinement exercise. No formal quantitative assessment of inter-rater reliability was performed. Therefore, the current work demonstrates conceptual and practical feasibility rather than statistical validation. Future prospective application in real-world guideline projects should assess the Framework’s feasibility, usability, inter-rater reliability, time burden, acceptability to guideline panels, impact on recommendation transparency, and potential areas for improvement.

Second, the Preconditions Checklist has so far been used only within the iterative refinement exercise. Its feasibility in real panel settings, including time burden and acceptability and its impact have not yet been evaluated. Further studies need to explore these aspects.

Third, when multiple forms of evidence are present, guideline developers must still exercise some judgement to determine the dominant evidence base. The Semmelweis Framework reduces the amount of discretionary judgement required by introducing explicit classes and labelling rules, but it cannot eliminate judgement entirely, and this therefore remains a potential limitation.

Finally, the Framework restricts the use of strong recommendations in Classes III and IV, where the evidence is non-comparative or primarily judgement based. This helps mitigate the problem of issuing strong recommendations on weak evidentiary foundations. However, the issue is not fully resolved, because this restriction does not apply to Classes I and II. Guideline panels may therefore still issue strong recommendations based on low or very low certainty evidence when the recommendation is grounded in direct or indirect comparative evidence. Extending the same restriction to Classes I and II would substantially constrain the ability of panels to issue recommendations within the standard GRADE structure and would make the Framework less compatible with existing GRADE-based processes. This limitation is therefore inherited from GRADE and is shared by the Semmelweis Framework.

## Conclusions

The Semmelweis Framework provides a structured approach for integrating empirical evidence and expert opinion in clinical practice guidelines. This is operationalised by classifying recommendations according to their evidentiary foundations and redistributing the roles traditionally grouped under “expert opinion” across explicitly defined recommendation classes, supported by a dedicated Checklist and avoiding the use of expert opinion as a generic recommendation label. The Framework is designed to serve as an umbrella structure for transparent and actionable recommendations, enabling guideline developers to support clinicians’ decisions even when evidence is limited.

## Supplementary Information

Additional file 1: Recommendations used in the iterative refinement exercise.

Additional file 2: The Preconditions Checklist.

Additional file 3: Frequently asked questions (FAQ).

Additional file 4: AI prompts.

## Data Availability

The Semmelweis Framework was developed using clinical recommendations from previously published guidelines, which are freely available and accessible. The complete list of recommendations, alongside the guidelines with their references is provided in the additional files. In addition, the two previous studies on which the Framework is founded are referenced in the main text, available as open access.

## Abbreviations

CoE: Certainty of Evidence
FAQ: Frequently Asked Questions
GRADE: The Grading of Recommendations, Assessment, Development and Evaluation
RoB: Risk of Bias
