# Supplementary material for: The Semmelweis Framework: structuring evidence foundations and expert opinion in clinical practice guidelines
Source: BMC Med Res Methodol. 2026 Jul 18;26:199. doi: 10.1186/s12874-026-02938-6 (PMC13540927; doi:10.1186/s12874-026-02938-6)
Supplement: Supplementary file 1 [file 12874_2026_2938_MOESM1_ESM.docx]

**Additional file 1 - Recommendations used in the iterative refinement exercise**

| **ID** | **Guideline** | **Year** | **Recommendation** |
| --- | --- | --- | --- |
| 1 | Martin-Loeches I, Torres A, Nagavci B, et al. ERS/ESICM/ESCMID/ALAT guidelines for the management of severe community-acquired pneumonia. Intensive Care Med. 2023;49(6):615-632. doi:10.1007/s00134-023-07033-8  (PILOT) | 2023 | We suggest the addition of macrolides, not fluoroquinolones, to beta-lactams as empirical antibiotic therapy in hospitalised patients with sCAP (conditional recommendation, very low quality of evidence). |
| 2 | Gossec L, Kerschbaumer A, Ferreira RJO, et al. EULAR recommendations for the management of psoriatic arthritis with pharmacological therapies: 2023 update. Ann Rheum Dis. 2024;83(6):706-719. Published 2024 May 15. doi:10.1136/ard-2024-225531  (PILOT) | 2024 | In patients in sustained remission, tapering of DMARDs may be considered. |
| 3 | Baughman RP, Valeyre D, Korsten P, et al. ERS clinical practice guidelines on treatment of sarcoidosis. Eur Respir J. 2021;58(6):2004079. Published 2021 Dec 16. doi:10.1183/13993003.04079-2020 | 2021 | For patients with cutaneous sarcoidosis and cosmetically important active skin lesions which cannot be controlled by local treatment, we suggest oral glucocorticoids be considered to reduce skin lesions. (Conditional recommendation, very low quality of evidence.) |
| 4 | Norris AH, Shrestha NK, Allison GM, et al. 2018 Infectious Diseases Society of America Clinical Practice Guideline for the Management of Outpatient Parenteral Antimicrobial Therapy. Clin Infect Dis. 2019;68(1):e1-e35. doi:10.1093/cid/ciy745 | 2019 | 6. In patients with no prior history of allergy to antimicrobials in the same class, the first dose of a new parenteral antimicrobial may be administered at home under the supervision of healthcare personnel who are qualified and equipped to respond to anaphylactic reactions (weak recommendation, very low-quality evidence). |
| 5 | Norris AH, Shrestha NK, Allison GM, et al. 2018 Infectious Diseases Society of America Clinical Practice Guideline for the Management of Outpatient Parenteral Antimicrobial Therapy. Clin Infect Dis. 2019;68(1):e1-e35. doi:10.1093/cid/ciy745 | 2019 | 9. For patients with advanced CKD requiring OPAT, a t-CVC is recommended rather than a PICC (strong recommendation, low-quality evidence). |
| 6 | Norris AH, Shrestha NK, Allison GM, et al. 2018 Infectious Diseases Society of America Clinical Practice Guideline for the Management of Outpatient Parenteral Antimicrobial Therapy. Clin Infect Dis. 2019;68(1):e1-e35. doi:10.1093/cid/ciy745 | 2019 | 15. Vancomycin blood levels should be measured regularly throughout the course of OPAT treatment (strong recommendation, very low-quality evidence). The optimal frequency of measurement is undefined, but the general practice in the setting of stable renal function is once weekly. |
| 7 | Johnson S, Lavergne V, Skinner AM, et al. Clinical Practice Guideline by the Infectious Diseases Society of America (IDSA) and Society for Healthcare Epidemiology of America (SHEA): 2021 Focused Update Guidelines on Management of Clostridioides difficile Infection in Adults. Clin Infect Dis. 2021;73(5):e1029-e1044. doi:10.1093/cid/ciab549 | 2021 | 1. For patients with an initial CDI episode, we suggest using fidaxomicin rather than a standard course of vancomycin (conditional recommendation, moderate certainty of evidence). |
| 8 | Johnson S, Lavergne V, Skinner AM, et al. Clinical Practice Guideline by the Infectious Diseases Society of America (IDSA) and Society for Healthcare Epidemiology of America (SHEA): 2021 Focused Update Guidelines on Management of Clostridioides difficile Infection in Adults. Clin Infect Dis. 2021;73(5):e1029-e1044. doi:10.1093/cid/ciab549 | 2021 | For patients with a recurrent CDI episode within the last 6 months, we suggest using bezlotoxumab as a co-intervention along with SOC antibiotics rather than SOC antibiotics alone (conditional recommendation, very low certainty of evidence). |
| 9 | Wahba A, Kunst G, De Somer F, et al. 2024 EACTS/EACTAIC/EBCP Guidelines on cardiopulmonary bypass in adult cardiac surgery. Interdiscip Cardiovasc Thorac Surg. 2025;40(2):ivaf002. doi:10.1093/icvts/ivaf002 | 2024 | The use of low-molecular-weight starches in priming solutions is not recommended. (Table 36) |
| 10 | Wahba A, Kunst G, De Somer F, et al. 2024 EACTS/EACTAIC/EBCP Guidelines on cardiopulmonary bypass in adult cardiac surgery. Interdiscip Cardiovasc Thorac Surg. 2025;40(2):ivaf002. doi:10.1093/icvts/ivaf002 | 2024 | RIPC should be considered to improve clinical outcomes and reduce myocardial and kidney injury in patients anaesthetized with volatile anaesthetic agents. (Table 21) |
| 11 | Chang W, Lee S, Kim YY, et al. Interpretation, Reporting, Imaging-Based Workups, and Surveillance of Incidentally Detected Gallbladder Polyps and Gallbladder Wall Thickening: 2025 Recommendations From the Korean Society of Abdominal Radiology. Korean J Radiol. 2025;26(2):102-134. doi:10.3348/kjr.2024.0914 | 2025 | S23-1: Follow-up is not required for ADM with typical imaging features S23-2: If GBC and ADM cannot be clearly differentiated, follow-up with US, CT, or MRI is recommended |
| 12 | Chang W, Lee S, Kim YY, et al. Interpretation, Reporting, Imaging-Based Workups, and Surveillance of Incidentally Detected Gallbladder Polyps and Gallbladder Wall Thickening: 2025 Recommendations From the Korean Society of Abdominal Radiology. Korean J Radiol. 2025;26(2):102-134. doi:10.3348/kjr.2024.0914 | 2025 | S8: US with a high-frequency transducer can be considered for further characterization of GBPs ≥6 mm detected on US |
| 13 | Ivani G, Suresh S, Ecoffey C, et al. The European Society of Regional Anaesthesia and Pain Therapy and the American Society of Regional Anesthesia and Pain Medicine Joint Committee Practice Advisory on Controversial Topics in Pediatric Regional Anesthesia. Reg Anesth Pain Med. 2015;40(5):526-532. doi:10.1097/AAP.0000000000000280 | 2015 | There are insufficient data in children to determine if using LOR to air or saline to detect needle entry into the epidural space will result in clinically significant differences regarding safety, accuracy, and subsequent efficacy of the injected LA (Evidence B3 and Evidence B4). Thus, both the aforementioned alternatives are acceptable if care is taken to keep the injected volume at a minimum. |
| 14 | Ivani G, Suresh S, Ecoffey C, et al. The European Society of Regional Anaesthesia and Pain Therapy and the American Society of Regional Anesthesia and Pain Medicine Joint Committee Practice Advisory on Controversial Topics in Pediatric Regional Anesthesia. Reg Anesth Pain Med. 2015;40(5):526-532. doi:10.1097/AAP.0000000000000280 | 2015 | Imaging modalities (ultrasound, fluoroscopy) may help to avoid or visualize accidental intravascular needle placement in peripheral blocks, but data are lacking in PRA to determine the value of these techniques (expert opinion). |
| 15 | WHO recommendations on maternal health guidelines approved by the WHO Guidelines Review Committee [Internet]. 2nd edition. Geneva: World Health Organization; 2025. Available from: https://www.ncbi.nlm.nih.gov/books/NBK615644/ | 2025 | Newborns without complications should be kept in skin-to-skin contact (SSC) with their mothers during the first hour after birth to prevent hypothermia and promote breastfeeding. |
| 16 | WHO recommendations on maternal health guidelines approved by the WHO Guidelines Review Committee [Internet]. 2nd edition. Geneva: World Health Organization; 2025. Available from: https://www.ncbi.nlm.nih.gov/books/NBK615644/ | 2025 | The use of oxytocin (10 international units [IU], intramuscular / intravenous) is recommended for the prevention of postpartum haemorrhage for all births. In situations where women giving birth vaginally already have intravenous access, the slow intravenous administration of 10 IU oxytocin is recommended in preference to intramuscular administration. |
| 17 | American Academy of Orthopaedic Surgeons Management of Carpal Tunnel Syndrome Evidence-Based Clinical Practice Guideline. https://www.aaos.org/cts2cpg Published May 18, 2024 | 2024 | Strong evidence suggests corticosteroid injection does not provide long-term improvement of carpal tunnel syndrome |
| 18 | American Academy of Orthopaedic Surgeons Management of Carpal Tunnel Syndrome Evidence-Based Clinical Practice Guideline. https://www.aaos.org/cts2cpg Published May 18, 2024 | 2024 | Strong evidence suggests that there is no difference in patient reported outcomes between a mini-open carpal tunnel release and an endoscopic carpal tunnel release |
| 19 | Kidney Disease: Improving Global Outcomes (KDIGO) ANCA Vasculitis Work Group. KDIGO 2024 Clinical Practice Guideline for the Management of Antineutrophil Cytoplasmic Antibody (ANCA)-Associated Vasculitis. Kidney Int. 2024;105(3S):S71-S116. doi:10.1016/j.kint.2023.10.008 | 2024 | Recommendation 9.3.2.1: We recommend maintenance therapy with either rituximab, or azathioprine and low-dose glucocorticoids after induction of remission (1C). |
| 20 | Kidney Disease: Improving Global Outcomes (KDIGO) ANCA Vasculitis Work Group. KDIGO 2024 Clinical Practice Guideline for the Management of Antineutrophil Cytoplasmic Antibody (ANCA)-Associated Vasculitis. Kidney Int. 2024;105(3S):S71-S116. doi:10.1016/j.kint.2023.10.008 | 2024 | Recommendation 9.3.1.1: We recommend that glucocorticoids in combination with rituximab or cyclophosphamide be used as initial treatment of new-onset AAV (1B). |
| 21 | Chadwick PR, Trainor E, Marsden GL, et al. Guidelines for the management of norovirus outbreaks in acute and community health and social care settings. J Hosp Infect. 2023;136:127-191. doi:10.1016/j.jhin.2023.01.017 | 2023 | GPP 7.1: Wherever possible, test all symptomatic patients to establish whether their symptoms are due to norovirus infection. |
| 22 | Chadwick PR, Trainor E, Marsden GL, et al. Guidelines for the management of norovirus outbreaks in acute and community health and social care settings. J Hosp Infect. 2023;136:127-191. doi:10.1016/j.jhin.2023.01.017 | 2023 | GPP 18.2: For occupied, shared patient areas or multi- occupancy rooms, undertake terminal cleaning a minimum of 72 h after symptoms in the last case of norovirus have resolved. |
| 23 | Beuschlein F, Else T, Bancos I, et al. European Society of Endocrinology and Endocrine Society Joint Clinical Guideline: Diagnosis and therapy of glucocorticoid-induced adrenal insufficiency. Eur J Endocrinol. 2024;190(5):G25-G51. doi:10.1093/ejendo/lvae029 | 2024 | R 2.1 We suggest not to taper glucocorticoids in patients on short-term glucocorticoid therapy of <3-4 weeks, irrespective of the dose. In these cases, glucocorticoids can be stopped without testing due to low concern for HPA axis suppression |
| 24 | Beuschlein F, Else T, Bancos I, et al. European Society of Endocrinology and Endocrine Society Joint Clinical Guideline: Diagnosis and therapy of glucocorticoid-induced adrenal insufficiency. Eur J Endocrinol. 2024;190(5):G25-G51. doi:10.1093/ejendo/lvae029 | 2024 | R 3.1 We recommend that patients with current or recent glucocorticoid use who did not undergo biochemical testing to rule out glucocorticoid-induced adrenal insufficiency should receive stress dose coverage when they are exposed to stress. |
| 25 | Lurie F, Malgor RD, Carman T, et al. The American Venous Forum, American Vein and Lymphatic Society and the Society for Vascular Medicine expert opinion consensus on lymphedema diagnosis and treatment. Phlebology. 2022;37(4):252-266. doi:10.1177/02683555211053532 | 2022 | Sequential pneumatic compression (SPC) should be recommended for lymphedema patients |
| 26 | Lurie F, Malgor RD, Carman T, et al. The American Venous Forum, American Vein and Lymphatic Society and the Society for Vascular Medicine expert opinion consensus on lymphedema diagnosis and treatment. Phlebology. 2022;37(4):252-266. doi:10.1177/02683555211053532 | 2022 | Surgery should be considered for patients with failed conservative treatment in higher than the second ISL stage |
| 27 | US Preventive Services Task Force, Davidson KW, Barry MJ, et al. Screening for Colorectal Cancer: US Preventive Services Task Force Recommendation Statement. JAMA. 2021;325(19):1965-1977. doi:10.1001/jama.2021.6238 | 2021 | The USPSTF recommends screening for colorectal cancer in all adults aged 50 to 75 years. |
| 28 | US Preventive Services Task Force, Davidson KW, Barry MJ, et al. Screening for Colorectal Cancer: US Preventive Services Task Force Recommendation Statement. JAMA. 2021;325(19):1965-1977. doi:10.1001/jama.2021.6238 | 2021 | The USPSTF recommends that clinicians selectively offer screening for colorectal cancer in adults aged 76 to 85 years. Evidence indicates that the net benefit of screening all persons in this age group is small. In determining whether this service is appropriate in individual cases, patients and clinicians should consider the patient's overall health, prior screening history, and preferences. |
| 29 | Fitridge R, Chuter V, Mills J, et al. The intersocietal IWGDF, ESVS, SVS guidelines on peripheral artery disease in people with diabetes and a foot ulcer. Diabetes Metab Res Rev. 2024;40(3):e3686. doi:10.1002/dmrr.3686 | 2023 | Recommendation 20. In a person with diabetes, peripheral artery disease, and a foot ulcer or gangrene undergoing an endovascular procedure, consider targeting the artery on angiography that supplies the anatomical region of the ulcer, when possible or practical. |
| 30 | Fitridge R, Chuter V, Mills J, et al. The intersocietal IWGDF, ESVS, SVS guidelines on peripheral artery disease in people with diabetes and a foot ulcer. Diabetes Metab Res Rev. 2024;40(3):e3686. doi:10.1002/dmrr.3686 | 2023 | Recommendation 15. In a person with diabetes and a foot ulcer, when the wound deteriorates or fails to significantly improve (e.g., a less than 50% reduction in wound area within four weeks) despite appropriate infection and glucose control, wound care, and offloading, reassess the vascular status and consult with a vascular specialist regarding possible revascularisation. |
| 31 | Del Rivero J, Perez K, Kennedy EB, et al. Systemic Therapy for Tumor Control in Metastatic Well-Differentiated Gastroenteropancreatic Neuroendocrine Tumors: ASCO Guideline. J Clin Oncol. 2023;41(32):5049-5067. doi:10.1200/JCO.23.01529 | 2023 | Recommendation 1.3. Patients should be assessed for SSTR positivity using SSTR positron emission tomography (PET) with 68 Ga-DOTA-peptides or 64 Cu-DOTATATE at baseline (Type: Informal consensus; benefits outweigh harms; Evidence quality: Low; Strength of recommendation: Strong). |
| 32 | Del Rivero J, Perez K, Kennedy EB, et al. Systemic Therapy for Tumor Control in Metastatic Well-Differentiated Gastroenteropancreatic Neuroendocrine Tumors: ASCO Guideline. J Clin Oncol. 2023;41(32):5049-5067. doi:10.1200/JCO.23.01529 | 2023 | Recommendation 2.3. Peptide receptor radionuclide therapy (PRRT) is recommended for patients with SSTR-positive metastatic G1-G2 GI-NETs that have progressed following treatment with SSAs (Type: Evidence based; benefits outweigh harms; Evidence quality: Moderate; Strength of recommendation: Weak). |
