# Supplementary material for: The Semmelweis Framework: structuring evidence foundations and expert opinion in clinical practice guidelines
Source: BMC Med Res Methodol. 2026 Jul 18;26:199. doi: 10.1186/s12874-026-02938-6 (PMC13540927; doi:10.1186/s12874-026-02938-6)
Supplement: Supplementary file 2 [file 12874_2026_2938_MOESM2_ESM.docx]

**Additional file 2 - Preconditions Checklist for recommendation Classes II, III and IV.**

*For the recommendation to be issued, all questions must be answered “Yes”. If any question is answered “No”, the recommendation should not be issued. Instead, a research recommendation may be more appropriate.*

*The completed checklist for each recommendation can be published in the guideline’s supplementary material.*

| **No.** | **Preconditions** | **Answer**  **(Yes/No)** | **Justification**  **(Briefly explain your answer)** |
| --- | --- | --- | --- |
| **Evidence** | | | |
| 1 | Were systematic searches conducted to identify relevant evidence? |  |  |
| 2 | Did the panel discuss the reasons why there is no (sufficient) direct evidence, including practical, ethical, or financial barriers to conducting relevant research? |  |  |
| **Necessity** | | | |
| 3 | Is issuing this recommendation necessary and justified to improve clinical decision-making despite the evidence gap? |  |  |
| 4 | Did the panel consider the important potential (direct and indirect) benefits and harms arising from this recommendation? |  |  |
| 5 | Is the rationale for issuing this recommendation (including key uncertainties and assumptions) documented in a way that is understandable and traceable for guideline users? |  |  |
| **Patient & Research Impact** | | | |
| 6 | Did the panel discuss potential acceptability and likely values/preferences of patients and other stakeholders based on available information? |  |  |
| 7 | Did the panel consider whether this recommendation could have different effects on specific subgroups (e.g., with certain comorbidities, socioeconomic status, etc.)? |  |  |
| 8 | Did the panel explicitly document key evidence gaps and research priorities to ensure that issuing this recommendation does not replace the need for further research? |  |  |
| **Panel Integrity** | | | |
| 9 | Did the panel include sufficient relevant clinical and methodological expertise to issue this recommendation despite the evidence gap? |  |  |
| 10 | Was the decision-making process conducted in a way that minimised undue influence by any individual and ensured balanced contribution across panel members? |  |  |
| 11 | Were potential conflicts of interest of all panel members disclosed, assessed, managed and documented in a way that makes undue influence on this recommendation unlikely? |  |  |
