# Supplementary material for: The Semmelweis Framework: structuring evidence foundations and expert opinion in clinical practice guidelines
Source: BMC Med Res Methodol. 2026 Jul 18;26:199. doi: 10.1186/s12874-026-02938-6 (PMC13540927; doi:10.1186/s12874-026-02938-6)
Supplement: Supplementary file 3 [file 12874_2026_2938_MOESM3_ESM.docx]

**Additional file 3: Frequently asked questions (FAQ) for adoption and implementation of the Semmelweis Framework**

| **No.** | **Question** | **Answer** |
| --- | --- | --- |
| 1 | Why does the Framework use “expert opinion” in the title if it advises against using the term? | The term “expert opinion” is used in the title because it is widely recognised in guideline methodology and helps readers immediately understand the topic the Framework addresses. At the same time, the Framework discourages using “expert opinion” as a vague, catch-all justification within recommendations. Instead, it operationalises what is often labelled “expert opinion” by distributing such situations across structured classes (II–IV), each with a defined evidentiary foundation and explicit safeguards and conditions for use. For the traditional scenario in which guidance is based primarily on clinical expertise in the absence of meaningful evidence, the Framework uses Class IV (“expert judgement”), which explicitly signals the nature of this foundation. This makes the role of expertise traceable, reduces ambiguity, and improves transparency for guideline users. |
| 2 | Is expert opinion the same as consensus? | No, they are different concepts. Expert opinion is the substance (clinical experience, mechanistic reasoning, interpretation of evidence, etc.) that fills an evidence gap. Consensus is the decision-making approach (e.g., discussion, Delphi, majority vote, etc.) used to agree on a recommendation. These two terms should not be used interchangeably. |
| 3 | How do the Semmelweis classes and their terminology relate to GRADE certainty of evidence and strength of recommendations? | They are separate dimensions. While the Semmelweis class describes the type and structure of the evidentiary foundation (direct vs indirect, comparative vs non-comparative, versus expert judgement), GRADE certainty, in contrast, reflects the confidence in the effect estimate for each outcome, based on risk of bias, inconsistency, indirectness, imprecision, and publication bias. Strength of recommendation (strong vs conditional) is then determined by the full GRADE process (including benefits and harms, values, feasibility, equity, etc.).  As a result, it is possible to have, for example, a Class I recommendation supported by randomized trials but with low certainty for a given outcome, or a Class II recommendation with moderate certainty for a specific outcome. Panels should therefore always report both the Semmelweis class and the GRADE certainty (see Table 1 for examples). |
| 4 | Which risk-of-bias (RoB) assessment tool should we use to assess the quality of studies? | The Semmelweis Framework does not suggest a specific RoB tool. Guideline-developing organisations should select tools according to their own methods manual and the study designs included (e.g. different tools for randomised trials, non-randomised comparative studies, diagnostic studies etc.). The key requirement is that validated, design-appropriate RoB tools are applied consistently across all recommendations. The Framework is compatible with any such tools. |
| 5 | What if the recommendation seems to be supported by evidence which fits in more than one class? | When evidence spans more than one class, panels should assign the class that reflects the dominant foundation that had the largest influence on the final judgement. For example, if direct comparative evidence exists but the recommendation was primarily driven by indirect evidence because the direct evidence was not applicable or was too uncertain, the recommendation should be classified according to that dominant driver.  If two classes contributed equally and the panel cannot reasonably distinguish which had the greater influence, the higher class may be chosen as the label for the recommendation. |
| 6 | How should we distinguish between an uncontrolled cohort and a case series when assigning classes? | In the Semmelweis Framework, uncontrolled cohorts are classified as Class III, whereas case series are classified as Class IV. The key distinction is the sampling frame and the presence of a denominator. In an uncontrolled cohort study, patients are sampled based on an exposure or membership in a clearly defined group (for example, all patients receiving a particular intervention over a specified period), and outcomes are assessed during follow-up. This design, at least in principle, allows calculation of the absolute risk for that exposed group. By contrast, a case series samples patients based on having the outcome (or disease), but without a well-defined source population of all exposed individuals. In such studies, the risk of outcome given exposure cannot be calculated.  *More info: Dekkers OM, Egger M, Altman DG, Vandenbroucke JP. Distinguishing case series from cohort studies. Ann Intern Med. 2012;156(1 Pt 1):37-40* |
| 7 | How do we classify evidence from a Systematic Review? | Systematic reviews do not automatically fall into a single class. A systematic review is a method of synthesis, not a study design itself. Therefore, the classification depends entirely on the underlying evidence included within the review.  Guideline panels must assess the primary studies synthesised in the review. The final class is determined by the highest-class evidence used to underpin the recommendation. For example, a systematic review of randomized trials would support a Class I or II recommendation, whereas a systematic review of case series would support a Class IV recommendation. |
| 8 | How does the Framework apply to Diagnostic Test Accuracy (DTA)? | The same principles apply. The classification depends on the study type and its directness to the patient-important outcomes:  Class I: Based on a direct comparative study that measures patient outcomes (e.g., RCTs or prospective comparative cohorts comparing test-treat strategies).  Class II: Based on a Diagnostic Test Accuracy (DTA) study. A DTA study is comparative (test vs. standard), but "accuracy" is a surrogate outcome. Using it to infer patient outcomes is a form of outcome indirectness, placing it in Class II.  Class III: Evidence from non-comparative but systematically collected datasets, e.g., single-arm prospective cohorts, registries without a concurrent reference standard.  Class IV: Case series, case reports, mechanistic or expert-opinion reasoning about tests (e.g., pathophysiological plausibility without data). |
| 9 | How does the Framework classify evidence based on modelling? | Modelling is an analytical approach, not a study design per se. When a model is used only to translate or project an existing effect estimate from a comparative study (e.g. RCT or observational study), the recommendation retains the classification of the underlying evidence—Class I if direct, Class II if indirect.  However, if the underlying evidence is non-comparative and the model is used to construct a counterfactual or effect estimate, the model becomes the primary foundation, and the recommendation should be classified as Class III.  The same applies when a model is the sole source of the effect estimate because adequate empirical studies are absent. In such cases, it functions as a form of mechanism-based reasoning, and the recommendation should be classified as Class IV. |
| 10 | How should we decide for or against a recommendation? | The Semmelweis Framework provides the structure for classifying and wording recommendations but does not determine the criteria or the system for the decision-making process. That decision is made by each guideline panel using the organisation’s own established process for weighing benefits and harms. The Semmelweis Framework is designed to be compatible with these approaches and does not replace them. |
| 11 | What is a “consideration” and how should clinicians interpret it compared with a conditional recommendation? | In the Semmelweis Framework, considerations are guidance statements used when recommendations rely mainly on non-comparative evidence (Class III) or expert judgement (Class IV).  Compared with a conditional recommendation, a consideration carries less prescriptive force. It does not define a default course of action for most patients. Instead, it flags an option or concern that clinicians should weigh carefully with patients, taking account of context, values, and local resources. Considerations should be interpreted as cautious, judgement-dependent guidance rather than as a standard of care. |
| 12 | Can we still issue strong recommendations when certainty of evidence is low or very low? | The Framework prevents strong recommendations when the evidentiary foundation is non-comparative (Class III) or based on expert judgement (Class IV).  For Classes I–II, the usual GRADE rules remain in place regarding the strength of recommendations (see GRADE guidance for more). |
| 13 | What if we do not issue a recommendation for a research question? | If no recommendation can be made, the authors must explicitly state the reason below. For example, “No recommendation could be made due to inconclusive evidence, very low certainty, or lack of consensus” etc. This helps guideline users understand right away why a recommendation was not issued.  Also, suggestions for further research may be issued. |
| 14 | What if we believe an expert opinion-based recommendation is necessary, but it does not meet all preconditions? | In such cases, a recommendation should not be issued until further evidence becomes available. Instead, a statement identifying the gap, flagging it as a research priority, may be used. |
| 15 | Can remarks be used to introduce additional guidance that is not part of the recommendation? | Remarks are used to supplement recommendations by adding contextual or practical guidance (e.g., scope, implementation considerations, monitoring, exceptions). However, remarks should not be used to introduce “hidden” recommendations. If a remark contains a clear action statement, it should be written as a separate recommendation and classified accordingly. Remarks must also be transparent: cite supporting evidence when evidence-based; if they reflect clinical expertise, this must be labelled explicitly in brackets (see Table 1 for examples). |
| 16 | Why should we label the reason for issuing a “no recommendation”? | Because “no recommendation” can mean fundamentally different things. Labelling the reason makes the decision transparent, helps users interpret uncertainty correctly, and prevents misinterpretation of “no recommendation” as evidence of no effect or no importance. It also documents whether the reason for not issuing a recommendation was evidence-related (e.g., very low certainty, conflicting results, or scarcity of evidence), process-related (e.g., no consensus in the panel), or contextual (e.g., substantial variability in values/preferences, feasibility constraints). Stating the reason highlights the key evidence gaps or decision barriers, helping to prioritise and design future research. |
| 17 | Can the Semmelweis Framework be used for public-health or health-system recommendations? | The current version of the Semmelweis Framework was primarily developed for clinical practice guidelines focused on individual patient care in treatment, diagnosis and prevention.  Public-health or system-level guidance often involves additional considerations (population-level effects, implementation logistics, policy constraints) that are outside the current scope.  However, until further specific guidance is published, organisations may freely adapt the current principles for public-health or health-system guidelines. |
| 18 | Does this Framework increase the workload for guideline developers? | As the Framework introduces a new method and additional rigour, it may initially be more time-consuming because of the need to complete the Preconditions Checklist, perform additional assessments, or hold further panel discussions. However, this additional workload is expected to be limited. These steps are required to ensure a reproducible and transparent method for utilising expert opinion. |
| 19 | Can we modify the Framework to fit our needs? | Since one of the aims of this Framework is also to provide consistency between organisations, it is advised that modifications are kept to a minimum. Organisations may adapt certain elements of the Framework while maintaining its principles and provided the four classes, expert opinion -preconditions, and phrasing rules remain intact. |
| 20 | How should we communicate Semmelweis-based recommendations to clinicians who are unfamiliar with the Framework? | The Semmelweis Framework uses practical terminology derived from the needs of guideline-developing organisations. Its terms are intended to be understandable to clinicians and lay users, including labels such as “direct evidence”, “indirect evidence”, “non-comparative evidence”, and “expert judgement”. |
| 21 | Should we publish Semmelweis classes and checklists in the main manuscript, or should they be supplementary? | For transparency, each recommendation’s class should be clearly displayed in the main guideline document, while the completed Preconditions Checklists for Classes II–IV should be published in the supplementary material. |
| 22 | How should we apply the Semmelweis Framework when updating an existing guideline? Can the recommendation class change over time? | Yes. The Semmelweis class should be re-assessed whenever a recommendation is updated. During an update, panels should identify the new evidence and reconsider the overall evidentiary foundation of the recommendation. If higher-class evidence becomes available (for example, moving from case series to an uncontrolled cohort, or from observational studies to randomized trials), the recommendation may shift from Class IV to III, or from Class III/II to Class I. |
