# Supplementary material for: The Semmelweis Framework: structuring evidence foundations and expert opinion in clinical practice guidelines
Source: BMC Med Res Methodol. 2026 Jul 18;26:199. doi: 10.1186/s12874-026-02938-6 (PMC13540927; doi:10.1186/s12874-026-02938-6)
Supplement: Supplementary file 4 [file 12874_2026_2938_MOESM4_ESM.docx]

**Additional file 4 – AI prompts used**

The main prompts used for preparing the Framework and writing the manuscript are listed below. Additional minor prompts of a similar nature were used throughout the writing process (e.g. to revise one specific sentence).

**ChatGPT (versions o3, 4.0, 5.1, 5.4, 5.5)**

1. “Find possible pitfalls or areas which are problematic in this framework which might hinder implementation”.
2. “Which text in the discussion I can shorten?”
3. “You are a methodologist, a reviewer for a renowned journal. Review this paper like a reviewer for the journal. Be impartial, concise and true.”
4. “Check the preconditions checklist for any inconsistencies, grammar issues, issues with text flow, or potential issues for misunderstanding the questions”.
5. “Check the comments with the authors' answers, and the revised manuscript. Check for mistakes or any misalignment between the two”.
6. “We need to use British spelling. Check this document”.

**Gemini (version 2.5 Pro)**

1. “You are a methodologist. Review this manuscript as you would when submitted to a renowned journal. Be clear and true”.
2. “Find possible pitfalls or areas which are problematic in this framework which might hinder implementation”.
3. “Check the preconditions checklist. Do you understand the reason why we created this checklist? Review current items. And make suggestions for adding new ones or deleting them”.
4. “Check for inconsistencies in names and abbreviations within the manuscript, and between documents”.
5. You are a graphic designer. Check and assess the design of this flowchart which will be published in a medical journal. Check if it’s clear, easy to read. And provide suggestions for improvement.
